# An evaluation of the innovative potentials of a HIV pilot exploring medical pluralism in rural South Africa

**DOI:** 10.1080/17290376.2018.1536560

**Published:** 2018-10-19

**Authors:** Christopher J. Burman

**Affiliations:** a The Community Engagement Unit, Rural Development and Innovation Hub, University of Limpopo, Polokwane, South Africa; b Turfloop Graduate School of Leadership, University of Limpopo, Polokwane, South Africa

**Keywords:** Adjacent possible, community-university partnership, innovation, HIV/makgoma conflation, opportunity vacuum model

## Abstract

This article reflects on an internal evaluation undertaken to estimate the potentials of a community-university pilot project to be developed into a bonafide innovation that can be applied at scale. The focus of the community-university partnership has been to reduce the unintended consequences of medical pluralism on the HIV and AIDS epidemic in Waterberg district, Limpopo Province, South Africa. Despite promising outputs from the partnership – including an increase in adherence to antiretroviral therapy and a reduction in stigma among traditionalists living with HIV – the partnership wished to establish whether further funding should be applied for to take the pilot from its current prototype status to a more established innovation. In order to evaluate the innovative potentials of the pilot, the opportunity vacuum model of innovation was adapted and applied. The findings indicate that (1) the application of the opportunity vacuum model of innovation to evaluate the potentials of the pilot to be developed into a bonafide innovation was fit for purpose and (2) the pilot contains the key ingredients that are associated with innovations in the making. The discussion reflects on the social potentials of the pilot to contribute to 90-90-90 from a global, national and local perspective. The reflection concludes by suggesting that the opportunity vacuum model of innovation is a versatile heuristic that could be applied in other contexts and the community-university pilot represents a nascent innovation which has sufficient potential to justify further development.

## Introduction

This article reflects on an evaluation of the innovative potentials of a community-university pilot project that has focused on the unintended consequences of medical pluralism on the trajectory of the HIV and AIDS epidemic in the Waterberg district, Limpopo Province, South Africa. The focus of the pilot was to reduce the ambiguities generated by localised beliefs in disease causation and subsequent treatment seeking practices in the context of HIV and AIDS. The ambiguities relating to disease causation and subsequent treatment seeking practices arose because the symptoms of some HIV-related co-infections are similar to symptoms associated with a traditional disease called makgoma (henceforth, the ‘HIV/makgoma conflation’). The HIV/makgoma conflation has been shown to contribute to delays in testing for HIV and to interrupt antiretroviral treatment (ART) (Shirindi & Makofane, 2015).

In response to this phenomenon, the community-university partnership designed and implemented a novel pilot (Burman & Aphane, 2016a). The most recent findings provide qualitative indicators that the pilot has contributed to an increase in adherence to ART and reduced stigma among traditionalists influenced by the HIV/makgoma conflation (Burman & Aphane, forthcoming 2018). Despite the localised success of the pilot, a question arose about whether, or not, it was justifiable to pursue further funding to increase the reach of the intervention. In order to generate indicators about the potential utility of the pilot to be developed into an authentic innovation (i.e. move from a prototype idea to a tangible service, or product), the opportunity vacuum model of innovation was adapted and applied. The findings from the evaluation indicate that the pilot does contain the principal ingredients associated with potential innovations and thus, it is justifiable to pursue further funding to increase the scale and reach of the intervention.

In order to present the findings of the evaluation, the following contextual background information is provided: (1) the changing HIV and AIDS environment; (2) medical pluralism and 90-90-90, including the HIV/makgoma conflation; (3) the community-university partnership, and (4) innovation theory, including the opportunity vacuum model of innovation, before the method is presented.

The methodological framework includes an adapted version of the opportunity vacuum model of innovation and a re-analysis of narrative data collected during group discussions with members of two support groups for people living with HIV in late 2016. The discussion provides a reflection on the findings from the perspective of: (1) global health policy recommendations; (2) the South African National Strategic Plan for HIV, TB and STIs 2017–2022 (NSP); (3) a local scale, (the lifeworlds of the people interviewed), and (4) the potentials contained within the findings to justify expanding the influence of the pilot. The article concludes by explaining that the opportunity vacuum model of innovation, when applied as a heuristic, was fit for the purpose it was used for and the community-university partnership will pursue opportunities to expand the pilot intervention.

## Background information

### The changing HIV and AIDS environment

In 2014, UNAIDS initiated a global campaign to end AIDS by 2030 (UNAIDS, 2014a, 2014b). South Africa was the first country to adopt the strategy and has committed to ensuring that ‘90% of all people living with HIV know their HIV status; 90% of all people with an HIV diagnosis receive sustained antiretroviral therapy, and 90% of all people receiving antiretroviral therapy achieve viral suppression’ (SANAC, 2017, p. xv). 90-90-90 is a biomedical strategy that is designed to ensure that the viral load of all people living with HIV is fully suppressed because a fully suppressed viral load significantly reduces mortality and morbidity associated with HIV infection (Teeraananchai, Kerr, Amin, Ruxrungtham, & Law, 2016) and minimises the risk of downstream HIV infection (Rodger, Bruun, & Cambiano, 2014). It has been estimated that the effectiveness of the second (treatment) and third (virally suppressed) ‘90’ has contributed to a 26% global decrease in annual HIV-related deaths since 2010 (UNAIDS, 2016). Notwithstanding this significant biomedical achievement, there are concerns that exclusively relying on a technical, biomedical solution to end AIDS comes with caveats because the epidemic is as much bio-social as it is biomedical (Vella, 2015).

The bio-social caveats were acknowledged by UNAIDS when they launched the 90-90-90 campaign. UNAIDS argued that in order to end AIDS by 2030 it would be necessary to (1) step beyond the parameters of ‘business as usual’ (UNAIDS, 2014b, p. 296); (2) appropriately and urgently respond to twelve marginalised, at risk, populations that face ‘complex life challenges, risks and obstacles on multiple fronts’ (UNAIDS, 2014b, p. 118), and (3) that there are different types of HIV and AIDS epidemics which vary considerably within and between countries and regions, and …  [s]uccess [in ending AIDS] relies on focusing on the locations and populations where risk is greatest’ (UNAIDS, 2014a, p. 18). The South African National AIDS Council (SANAC) takes a similar view, arguing that the NSP encourages the ‘roll-out of innovative [bio-social] approaches to increase treatment uptake and improve treatment outcomes’ (SANAC, 2017, p. 21) and to identify and respond to other ‘groups left behind’ (SANAC, 2017, p. xii).

Despite action to minimise the adverse influence of some bio-social factors, examples persist. For example, in South Africa, one bio-social caveat is that of the 3.7 million who have been initiated on ART, non-adherence ranges from 21.4% (North West Province) to 34.4% (Limpopo Province) at 12 months, representing a national average of 27%, which increases to almost 50% at five years (SANAC, 2016, p. 39). A second, discrete caveat in South Africa is the broader – and empirically ambiguous – influence of medical pluralism on 90-90-90.

## Medical pluralism and 90-90-90

Medical pluralism can be defined as the co-existence of two or more health care systems within a particular environment which is a common phenomenon ‘found in almost every country in the world’ (WHO, 2013, p. 7). Typically, different health care systems have different ideal-type mental models relating to disease causation and subsequent treatment seeking practices (Dubois, 1961). For example, the ‘biomedical model presupposes that there are specific diseases, each associated with a specific biological process, and that the *cause is biologically specific*’. On the other hand, African traditional medicine models are based on beliefs that presuppose that ‘[i]llness[es] ha[ve] *many causes* …  which include disturbance of social relationships, spirits, supernatural forces, and deliberate poisoning’ (emphasis added, Ibeneme, Eni, Ezuma, & Fortwengel, 2017, pp. 14 & 16). In naturalistic situations, these ideal-types manifest through social practices associated with different ‘street level epistemology[ies] of trust’ relating to the degree of trust that individuals, or social collectives, have in either/or the ideal-type mental models (Rubincam, 2015, p. 3).

**Figure 1.**
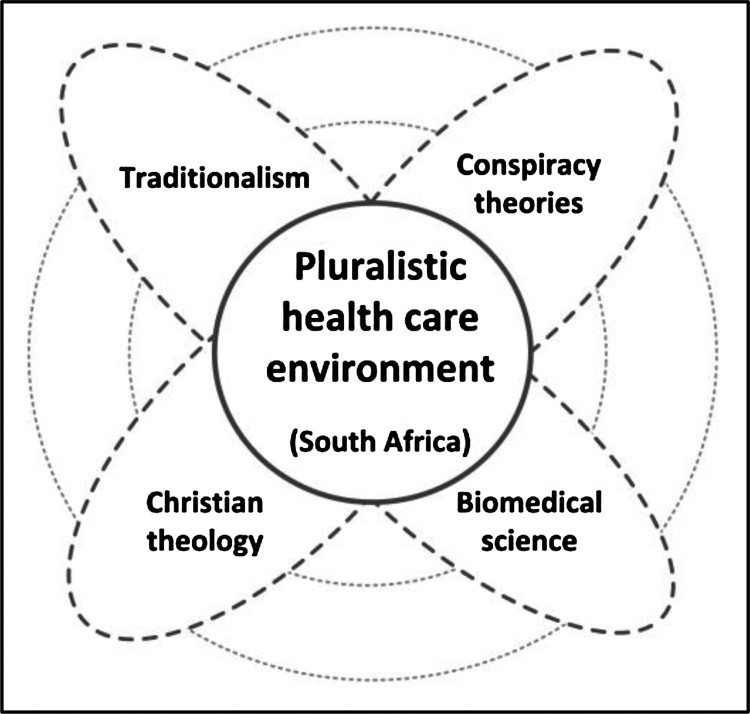
The pluralistic health care environment in South Africa. Source: Author’s contribution.

In South Africa, the pluralistic health care environment is influenced by traditionalist beliefs (people who believe in the effectiveness of African traditional medicine, McFarlane, 2015); conspiracy theories (people who believe that HIV and AIDS was invented by whites as a from of racial control over blacks, Nattrass, 2013) and Christian theology (people who believe that HIV and AIDS was created by God to punish immoral sexual behaviour, Murthy, 2016), all of which converge with the biomedical health care system in diverse ways, Figure 1.

The narrative material that is evaluated using the opportunity vacuum heuristic below, relates to the convergence of the traditional and biomedical health care systems in South Africa.

### The pluralistic health care environment in South Africa

In 2002, the World Health Organisation (WHO) stated that 80% of sub-Saharan Africans used traditional medicine, or a combination of traditional and biomedical medicine (WHO, 2002). Subsequent data indicates that ‘the ratio of traditional healers to population in Africa is 1:500, whereas the ratio of medical doctors to population is 1:40 000. [This means that] for millions of people in rural areas, native [traditional] healers therefore remain their health providers’ (WHO, 2013, p. 19; citing Abdullahi, 2011).

In South Africa, evidence relating to the use of traditional medicine indicates that whilst the traditional health care sector remains a ‘valid and necessary’ cultural component of the health care system (McFarlane, 2015, p. 67), the influence of traditional health care practices is declining (Flint & Payne, 2013). Case studies from Limpopo and Kwa-Zulu Natal Provinces, South Africa, corroborate this with figures that are closer to 50% in rural areas (Friend-du Preez & Peltzer, 2010; Rankoana, 2012). Despite the decline in the use of traditional medicine, it has been estimated that there are approximately 200,000 registered traditional health practitioners treating approximately 27 million people in South Africa (Street, 2016) which far exceeds the ratio of biomedical health care practitioners to population (De Roubaix, 2016). In the context of HIV, it has been demonstrated that two unintended consequences of the convergence of the traditional and biomedical health care systems, are delays in testing and interruption of treatment (Moshabela et al., 2017).

The unintended consequences of medical pluralism on the HIV and AIDS epidemic has been documented for over a decade (for one example of the earliest reports, see Kalichman & Simbayi, 2004). It is now being argued that the end of AIDS in sub-Saharan Africa cannot be achieved without acknowledging and responding to both traditional health care practices (Leclerc-Madlala, Green, & Hallin, 2016) and the convergences of different health care systems because many traditionalists are ‘able to straddle two health-worlds simultaneously’ (Moshabela et al., 2017, p. 4). The normalcy of ‘straddling’ is emphasised by SANAC (2016, p. 77) whose representatives from Limpopo ‘highlighted poor adherence to ART due to the use of alternative or traditional medicines’. One example of how traditional health care practices converges with the HIV and AIDS epidemic in South Africa is represented by a disease that Sotho-Tswana language users call makgoma.

#### Makgoma

Makgoma is a disease that Sotho-Tswana speaking traditionalists associate with ‘ritual taboo violations’ (Makgahlela & Sodi, 2016, p. 543). Ritual taboo violations represents a social practice that occurs when a person fails to conduct a specific ritual to overcome a particular cultural transgression such as ‘sleeping with a woman following an abortion’ (Mabunda, Khoza, Van den Borne, & Lebese, 2016, p. 5). Failure to rectify the transgression can result in physical symptoms that represent ‘contamination’ of the body which is considered to be a ‘taboo’ among traditionalists (Shirindi & Makofane, 2015, p. 942). It is possible to treat the contamination by undertaking remedial ‘cleansing rituals’ (Niehaus, 2013, p. 135). Without the culturally prescribed remedial cleansing ritual/s the offender risks the contamination developing into makgoma which can be transmitted to others, and/or result ‘in the patient fading (dying) away’ (parenthesis in original, Mönnig, 1978, p. 67). The symptoms of makgoma are similar to symptoms associated with HIV co-infections, such as tuberculosis (Mabunda et al., 2016).

A report in a South African newspaper, The Sowetan, relating to the murder of Orlando Pirates goalkeeper Senzo Meyiwa, emphasises how the contamination associated with makgoma converges with the HIV and AIDS epidemic:

A nyatsi definitely needs to be cleansed after the passing of her married lover. If not, not only is she in immediate danger, but she puts whoever else she comes in sexual contact with in danger too. Culturally, we believe that you have makgoma [dirty blood] if your lover passes away, and if you don’t get proper cleansing and rituals, anyone you sleep with will get so sick, and even have the same symptoms as someone with full-blown AIDS. So it is imperative to follow the correct rituals’. (parenthesis in original, Disetlhe, 2014, quoting a representative from the National House of Traditional Leaders)

**Figure 2.**
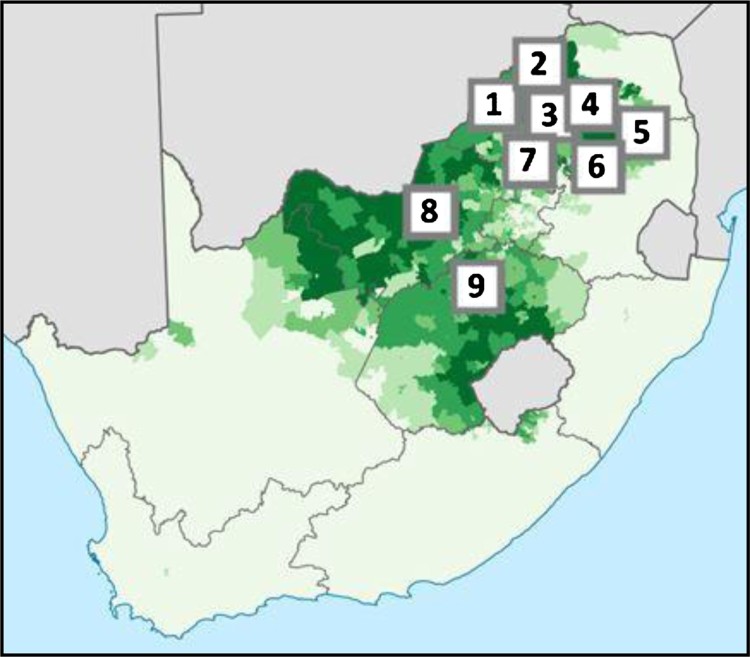
A Sotho-Tswana language map, South Africa, with some examples of where makgoma has been reported on in academic writing. Source: Adapted from the 2011 census broken down to ward level, available at: https://en.wikipedia.org/wiki/Sotho-Tswana_peoples#/media/File:South_Africa_2011_Sotho-Tswana_speakers_proportion_map.svg. Using Wikipedia as a source is not generally preferred because Wikipedia is not peer reviewed. However, in this instance it is used because the map enables an important concept to be provided and no other source has been identified.

Makgoma has been documented for four decades (for an example of one of the earliest reports see Mönnig, 1978). Figure 2 provides an overview of where makgoma has been recently reported on in academic writing.

Geographically, most documented reports relating to makgoma are reflected in case materials from Limpopo Province, but there are outlying reports in other provinces (North West, Gauteng, Free State and Mpumalanga Provinces). In northern and central South Africa, the Sotho-Tswana language has widespread, yet variable, levels of coverage. The darker shaded area in Figure 2 represents 80–100% coverage, with the lightest area representing 0–20%. By cross-referencing the language map with academic literature relating to makgoma, it has been possible to provide an indicator of the presence that makgoma has within this geographical language use area. The numbers on the map represent different areas where academic literature has reported makgoma and the associated influence it has on social practices (Table 1).

**Table 1.** Legend for the numbers shown in Figure 1.

Legend number	Influence of makgoma on social practices	Province	Area
1	Infant death and ceremonial burial rituals (Boeyens, van der Ryst, Coetzee, Steyn, & Loots, 2009)	Limpopo	Waterberg District
2	Craft art (Joubert, 2009)	Limpopo	Blouberg area in the Capricorn District
3	HIV and AIDS (Shirindi & Makofane, 2015) and many postgraduate degree dissertations from the Department of Nursing at the University of Limpopo, and other accredited articles relating to health care	Limpopo	Capricorn District
4	Medicinal plant use (Chauke, Shai, Mogale, Tshisikhawe, & Mokgotho, 2015)	Limpopo	Mopani District
5	Ritual taboos (Niehaus, 2013)	Limpopo	Bushbuckridge District
6	Tuberculosis & treatment seeking behaviour (Mabunda et al., 2016)	Limpopo	Sekhukhune, Lepelle-Nkumpi and Sekhukhune Districts
7	HIV and AIDS (Sebata, 2015)	North West	Mahikeng Municipality
8	Stigma, HIV and taverns (Niehaus, 2007)	Gauteng	Rustenberg
9	Adherence to ART (Serekoane, 2010)	Free State	Xhariep District

Source: Author’s contribution.

Figure 2 and Table 1 provide a generic overview of both the widespread coverage of makgoma and the variable influences the concept has on social practices. Table 2 provides an estimate of how many people living with HIV that the HIV/makgoma conflation may influence.

**Table 2.** An estimate of how many people living with HIV the HIV/makgoma conflation may influence.

Province	HIV prevalence by province, South Africa 2012 (Shisana et al., 2014)	Percentage distribution of the projected provincial share of the total population, 2002–2016 (STATS-SA, 2016)	Total living in that province (adapted from STATS-SA, 2016)	Total living with HIV in that province	50% of people living with HIV may be influenced by traditional values
	%	%	*n*=	*n*=	*n*=
Free State	14	5.1	2851410	399197	199599
Gauteng	12.4	24.1	13474310	1670814	835407
Limpopo	9.2	10.4	5814640	534947	267473
North West	13.3	6.8	3801880	505650	252825
Total	48.9	46.4	25942240	3110609	1555304

Source: Author’s contribution.

Table 2 provides an estimate of the number of people living with HIV that the HIV/makgoma conflation may influence. The analysis suggests that as many as 1.5 million people living with HIV may be influenced by traditional health care beliefs and practices which represent approximately 22% of the total number of people known to be living with HIV in South Africa. However, due to the detailed colour coding in Figure 1, this estimate is imprecise.

It should also be noted that traditionalism is not confined to the geographical coverage in Figure 2. For example, in Kwa-Zulu Natal Province, traditionalism has been identified as a ‘predictor’ of non-adherence to ART (Pantelic et al., 2015, p. 137) and there have been reports from across eastern and southern Africa that traditionalism generates ‘bottlenecks’ in the HIV continuum of care (Moshabela et al., 2017, p. 1). Despite awareness of the unintended, yet adverse, influence of traditional health care practices, including makgoma, on the trajectory of the HIV epidemic, the only reports relating to strategies to reduce this influence have been limited to *ad hoc* and short-lived interventions (Leclerc-Madlala et al., 2016).

### The community-university partnership

The partnership in this pilot is between the Community Engagement Unit, University of Limpopo (UL) and a non-profit organisation called the Waterberg Welfare Society (WWS, 2017). The primary mission of WWS is to promote HIV and AIDS wellness among marginalised communities in the rural Waterberg district of the Limpopo Province. One of their core mandates includes outreach work with deep rural communities primarily in the Waterberg district. Waterberg district has one of the higher levels of economic inequality in South Africa (Mostert & Van Heerden, 2015) and it is estimated that just under 30% of women attending an antenatal clinic in the district are HIV positive (Shisana et al., 2014).

The pilot was activated in the latter part of 2013 and is on-going. Ethical clearance was granted by the Turfloop Research and Ethics Committee, University of Limpopo, prior to implementation (clearance number TREC/FSA/16/2013: IR). Standard research ethics procedures have been complied with throughout. The drivers of the pilot have been UL’s Community Engagement Unit and WWS’ Education and Awareness Department (EAD). The EAD is comprised of two female nurses, a male school peer educator and a female child support worker. Their work includes facilitating support group meetings for people who are living with HIV in outlying communities. During the pilot the EAD were working with six support groups with a total membership of 187, of which three quarters were female (*n* = 128). Tat that time, the EAD aimed to meet with each support group on a monthly basis.

#### Implementation of the pilot

In the early phases of the pilot, the partnership agreed that the primary focus would be to identify and develop strategies to update WWS’ HIV and AIDS related wellness messaging. At that time the Abstain, Be faithful, Condomise (ABC) strategy had been officially phased out by the NDoH in favour of the 90-90-90 strategy. However, the opinions of WWS senior management were that the implications of 90-90-90 were not fully understood by both their own personnel and their neighbouring communities. In order to overcome this it was decided that it was necessary to train WWS personnel in the biomedical logic of 90-90-90 and then identify which parts of the biomedical logic could potentially be applied within WWS’ work environment (Burman & Aphane, 2016a).

The partnership decided that it was necessary to identify training that would enable people in the Waterberg district to make sense of the 90-90-90 strategy. The training that was chosen was called ‘A-3B-4C-T’ (details are available through The HIV/AIDS Communication and Media Network, 2014). The training took place at the WWS offices in 2014 and included senior members of WWS from the Child Disclosure Support Department (*n* = 5), Education and Awareness Department (*n* = 6), Treatment and Care Department (*n* = 2), the Department of Gender Based Programmes (*n* = 3) and Youth Environmental Services (*n* = 6).

Prior to the training two agreements were made by the community-university partnership. The first agreement was that the WWS participants would share the learning from the training with their departmental colleagues and then decide which aspects of the training could have utility in their different fields of work. The emphasis of the agreement was that it was the responsibility of each department to make decisions about the potential utility of the different components of training. Part of this agreement emphasised that there was no obligation to use any of the learning from training if it was deemed inappropriate for their particular working environment.

The second agreement was that each department would be responsible to begin using the learning from the training in ways that made sense in their working environments. The conditions attached to the process of integrating the learning included (1) that additional resources were not available for materials because the integration was considered to be in its infancy and, thus, funds would not be released until there was evidence of utility, and (2) that the integration processes that were adopted had to be ethically sound.

During the subsequent months the university monitored the integration process but did not influence the process of integration. The underlying logic being that WWS personnel were the experts in that field, thus it was inappropriate for the university personnel to interfere. After twelve months a two day synthesis of the integration efforts was undertaken by the partnership. At this point the university analysed the findings of the integration process using a thematic content analysis technique augmented by the Causal Layered Analysis (CLA) technique (Burman & Aphane, 2016b).

The analysis indicated that the EAD’s approach of integrating concepts from the training into their role plays, critical group and individual discussions with support group members and sharing this information with nurses working in local clinics that they collaborated with was demonstrating utility. It was then agreed by the partnership that three concepts would be focused on as action themes and that the EAD would take a lead in expanding the influence of the action themes within their outreach and wellness education activities (primarily role plays, critical group and individual discussions with support group members and sharing this information with nurses working in local clinics that they collaborated with).

#### The action themes applied during the pilot

Three action themes were agreed on by the partnership. The action themes include: the ‘origins of HIV’, the ‘viral load’ and the conceptualisation of the perception of ‘HIV as a death sentence to a chronic illness’ (Burman & Aphane, 2016a, p. 84).

The ‘origins of HIV’ action theme relates to the biomedical explanation of the origins of HIV which is called zoonosis (for an overview of this process in central Africa at the beginning of the last century see Sharp & Hahn, 2011). This action theme is relevant because some traditionalists, influenced by the HIV/makgoma conflation confuse the symptoms of an HIV co-infection with a ritual taboo violation and consequently seek treatment from a traditional health practitioner, rather than treatment from a biomedical health care provider.

The viral load action theme was considered relevant because it is now recommended that a person living with HIV regularly monitors their viral load as a mechanism to maintain good health (SANAC, 2017). Introducing the importance of the viral load into the educational packages delivered by the EAD was relevant to WWS’ clients because the relationship between the viral load and wellness was inadequately recognised by the majority of the support group members.

The final action theme was HIV being a chronic condition, not a death sentence. This action theme was considered relevant because there was a common belief by the support group’ membership that HIV is a death sentence, despite scientific evidence to the contrary (SANAC, 2017).The combination of the three action themes have been integrated into the educational packages used by the EAD during monthly support group meetings since the first quarter of 2016

The influence of the action themes on the 90-90-90 goals have been the focus of subsequent monitoring and evaluation. The inclusion of the three action themes into the educational packages has had most impact among traditionalists living with HIV. The outputs include (1) reducing the HIV/makgoma conflation by facilitating a distinction between the cause (origin) of HIV and the cause of makgoma; (2) increased adherence to ART, and (3) a reduction in stigma (Burman & Aphane, forthcoming 2018). Both (2) and (3) of these findings are priority areas identified in the NSP (SANAC, 2017).

## Innovation theory and the adjacent possible

The process of innovation – the generation of a novel, intangible idea and the subsequent development of the intangible concept into a tangible artefact – has a dynamic history (McGowan & Westley, 2015). One recent example of this dynamism, influenced by evolutionary theorist Kauffman (2000), is a concept called the adjacent possible.

### The adjacent possible – transposed into innovation theory

From an evolutionary perspective, the adjacent possible is a critique of the Darwinian argument that species develop primarily through relatively passive adaptation within a changing environment. Proponents of the adjacent possible theorem argue that not only do species adapt to environmental conditions, they also pro-actively seek out new evolutionary spaces which could provide conditions which might be exploitable. Having identified a potential new opportunity, the species begins by exploring the space and, if appropriate, inhabiting the new space. Having established a habitable niche within a new environment, species’ further development is influenced by the learning whilst adapting to, and exploiting, novel opportunities within the new environment – as well as the impact that species has on the host environment. The interdependencies between both the species and the habitus can produce an altered context, which may generate unexplored future potentials for further exploration (Tria, Loreto, Servedio, & Strogatz, 2014) Kauffman (2000, p. 207). called this process ‘expanding the adjacent possible’.

The adjacent possible has been drawn into social innovation theory because of the similarities between species’ evolutionary potentials and anthropogenic innovation. The similarities include (1) innovations being prompted by a deficiency, or inefficiency, within an existing habitus; (2) proactive exploration for an improved idea, or opportunity, to reduce, or overcome, the deficit situation, and (3) low-to-medium risk exploratory steps within the new environment to evaluate the potentials within it, prior to inhabiting it (Johnson, 2010).

From an anthropogenic perspective, the adjacent possible is commonly referred to as ‘the set of possibilities that are one step away from what actually exists’ (Gravino, Monechi, Servedio, Tria, & Loreto, 2016, p. 115). Exploratory journeys into the adjacent possible are typically creative, complex, iterative processes which may result in (1) variable degrees of emergent success; (2) generating serendipitous opportunities, and (3) failure (Gravino et al., 2016). One recent application of the adjacent possible within the field of micro-economics is called the ‘opportunity vacuum model’ (Planing, 2017, p. 1). This conceptualisation is described below because an adapted version is used to analyse the potentials of the community-university pilot to be developed into a more tangible innovation that could be replicated in other parts of South Africa.

## The opportunity vacuum model of innovation

**Figure 3.**
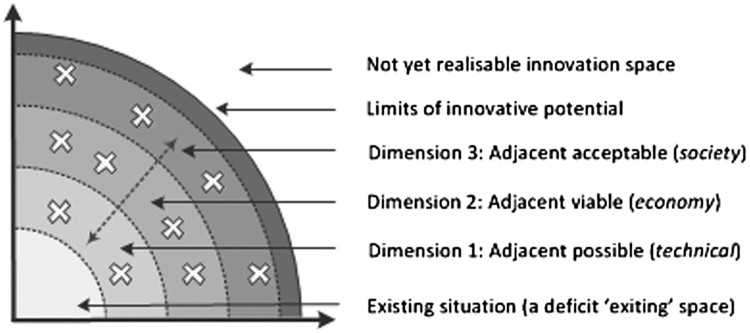
The vacuum model of anthropogenic innovation. Source: Adapted from Planing (2017, pp. 7–13).

The opportunity vacuum model is a heuristic that focuses on innovation processes at a micro-level. At the micro-level, the role of the individual in innovation has historically been associated with rare moments of genius or serendipity (Roberts, 1989). However, empirical evidence indicates that the lone genius is rarely the driver of innovation. The driver of innovation is now attributed as much to the individual as it is to the environmental conditions that enables new connections to develop between existing phenomena (Csikszentmihalyi, 2014). At a cognitive level, this literally means the emergence of unusual and/or unexplored neuronal networks, which are typically catalysed by awareness of a deficit situation, that are then developed into more concrete connections which have the potentials to generate the basis of tangible innovations (Johnson, 2010). In other words: most innovations ‘are built on *exiting* ideas [prompted by a perceived, or real, deficit within an existing habitus] but [are] modified by subtraction, division, merging, or multiplication’ of these ideas into potential innovations which are influenced by system-wide constraints and opportunities (emphasis added, Planing, 2017, p. 6) Planing (2017, p. 7). labels these systemic potentials as (1) the ‘adjacent possible (technical)’; (2) the ‘adjacent viable (economy)’, and (3) the ‘adjacent acceptable (society)’ which are described below, also see Figure 3.

### Dimension 1: adjacent possible (technical)

Technology – the application of scientific knowledge for practical purposes – has undoubtedly been an enabler of innovation. As one technology is developed it begins to open doors to new versions of (1) the same generic technological potentials being improved into slightly altered forms (the personal computer opened doors to laptops and IPads) and (2) qualitatively different innovations (the personal computer enabled the widespread use of the internet, Facebook and YouTube, representing a ‘disproportionately major consequences’).

### Dimension 2: adjacent viable (economy)

For the potentials of technological possibilities to be realised requires economic viability. Typically, a household innovation that is launched on to the market is initially considered a luxury item that is unaffordable to the majority (for example, the earliest home computers). With time, economies of scale and technological improvements, the cost may be sufficiently reduced for the item to become more affordable to a wider market. Without the affordability factor the innovation will invariably remain restricted to the wealthier segments of society.

### Dimension 3: adjacent acceptable (society)

The uptake of new technologies is invariably mediated by subjective societal preferences. This decision making is, more-often-than-not, highly contextualised, defies the belief that consumers consistently maximise utility and typically requires idiosyncratic time frames. This latter point is underscored by Rogers (2003, p. 436) who argued that ‘[c]hanging people’s customs [during processes of innovation] is an even more delicate responsibility than surgery in many cases’.

Planing (2017) provides extensive evidence to support the argument that for an intangible idea to be developed into a fully-fledged innovation by an artisan requires negotiation of the three dimensions described above, Figure 3.

Figure 3 represents the journey, represented by the diagonal dotted line, which an intangible idea that is generated in an ‘exiting’, deficit space has to travel if it is to become a tangible artefact that is adopted at scale. Planing (2017) emphasises that the journey is typically an iterative process, punctuated by complex feedback loops. He also emphasises that the three dimensions represent an interdependent whole which are separated for the sake of explaining the model. In reality, the iterations between, and within, the three dimensions are interconnected and represent the dispositional conditions which both enable and constrain the potentials of the transition from intangible idea to tangible artefact. The opportunity vacuum model is used as the basis for the methodology.

## Method

In late 2016, group discussions were undertaken with members of two support groups (*n* = 65) that the EAD work with in the rural villages of Mokamole and Lyden in the Mogalakwena Municipality, Waterberg district. The selection criteria for the purposefully sampled participants required that they were aged 18 years or above and had been a member of a support group that the EAD was working with for a minimum of two years. The purpose of the group discussions was to evaluate the effectiveness of the action themes in relation to the support group members’ treatment seeking practices (Burman & Aphane, forthcoming 2018).

The group discussion method was used because (1) the support group members were familiar to each other prior to the interviews (focus group discussions are between people who are not known to each other prior to the discussion); (2) homogeneous group discussions require people who are ‘comparable in the essential dimensions related to the research question and have a similar background’ (Flick, 2009, p. 196), and (3) the group discussion method enables qualitative data collection which generates insights into the way that ‘opinions are produced, expressed, and exchanged in everyday life’ (Flick, 2009, p. 197). The narrative data has been re-analysed from the perspective of evaluating the potentials of the pilot to be further developed into a viable innovation that can be applied at scale.

### Data collection

Five face-to-face, dual moderated, semi-structured homogenous group discussions of approximately one and a half hour duration were undertaken with groups comprised of up to a maximum of ten, mixed gender participants.. Two facilitators, one from WWS and one from the university, managed all of the discussions as both were fluent in Sotho-Tswana and English. The discussions used both local languages (Sotho-Tswana) and English. The topic guide included (1) the influence of traditionalism on the participants’ treatment seeking practices; (2) the influence of makgoma, both within their communities and on their treatment seeking practices; (3) the influence of the three action themes on their treatment seeking practices, and (4) an open session to enable any other information that the participants wished to share to be captured.

### Data analysis

The group discussions were recorded and then transcribed verbatim before being translated into English and back translated. The translated narratives were coded using a thematic analysis method (Vaismoradi, Jones, Turunen, & Snelgrove, 2016). The thematic codes that were applied were the different dimensions of the opportunity vacuum model proposed by Planing (2017).

In this instance the opportunity vacuum dimensions have been slightly modified in the following way: (1) the adjacent possible has been re-labelled the adjacent feasible because (a) the adjacent possible is now a generic expression that could lead to confusions among people already familiar with the expression adjacent possible and (b), the skills that the EAD applied to integrate the three action themes were not, strictly speaking, technological. The other dimension that has been altered is Dimension 3: the adjacent acceptable (society) because in this instance there are different scales that were relevant to the purpose of the evaluation. Consequently, Dimension 3 has been split into four sub-dimensions: 3.1, global scale (global health policy recommendations); 3.2, national scale (NSP); 3.3, local scale (lifeworlds of the group discussion participants) and 3.4, expanding the influence of the action themes. Nvivo, version 10, was used to facilitate the analysis. The findings were then discussed with the team leader of the EAD in order to ensure a measure of trustworthiness from the perspective of both the community-university partners.

## Findings

The analysis provides insights into two of the opportunity vacuum model components including (1) the ‘exiting’, deficit situation from which the potential innovation is emerging and (2) Dimension 3, the ‘adjacent acceptable (society)’. Secondary sources are used to provide additional insights into the potentials of the pilot to be developed into a tangible innovation.

The findings have been split into two sections. The first relates to the ‘exiting’ space theme and emergent categories, Table 3, followed by the three dimensions of the opportunity vacuum model and associated categories, Table 4.

**Table 3.** The ‘exiting’, deficit situation theme.

Categories	Examples from the transcribed narrative
The HIV/makgoma conflation (*n* = 21)	*When people are infected and sick, they still come with the explanation that the ancestors are calling them. It helps us* [*in the Support Group*] *to understand that HIV is different from makgoma*.
Stigma from the community (*n* = 15)	*We faced a lot of stigma and we did not know how to respond better to it. People still don’t believe HIV exists, they think we have dirty blood* [*makgoma*] *and we need a good traditional healer to help us.*
Internalised stigma (*n* = 13)	*We were shameful of ourselves and felt like culturally we did something wrong*.
Denialism that HIV exists (*n* = 12)	*It can be hard for people living with HIV to accept their HIV status. People are dying because they refuse to go to the clinic because they believe it is makgoma. Some do not even believe that HIV is real and can be treated properly at the clinic*.
Poor relationship between clinic staff and people living with HIV (*n* = 5)	*Nurses have changed their attitude towards us because we no longer miss our clinic appointments*.
Health policy: The first ‘90’ – testing, (*n* = 4)	*Nurses have been to schools and wait for people at street corners encouraging them to test for HIV. WWS have conducted HIV education and awareness events* – *but still people refuse to test because they think the sickness is makgoma that the traditional healer can cure.*
Health policy: The second ‘90’ – initiating treatment, (*n* = 3)	*They claim that in our tradition there is makgoma, not HIV. This causes a lot of confusion because some traditionalists don’t want to take treatment* because they don’t believe HIV exists.
Health policy: The third ‘90’ – achieving and maintaining a fully suppressed viral load	N/A

Source: Author’s contribution.

**Table 4.** Coding that relates to Planing’s (2017) opportunity vacuum model of innovation.

Theme	Categories	Examples from primary (narrative) or secondary sources
Dimension 1: Adjacent feasible (technology) – (*n* = 3)	The integration of the action themes into the EAD’s educational packages	The EAD integrated the action themes in late 2015 and they continue to use them in their outreach educational packages .
		*I have now accepted without any fear that I am HIV positive. This was because of the way WWS explained HIV and the viral load using roll plays helped us to understand what happens in our body with HIV and why we should take treatment* .
Dimension 2: Adjacent viable (economy)	Integration and no additional costs	The start-up costs to integrate the action themes into existing educational materials were subsidised through the research grant but there are now no additional costs. Further details have been reported on by Burman et al. .
Dimension 3.1: Adjacent acceptable – global scale (global health policy recommendations)	Health policy: the first ‘90’ – testing. Explanations as to why traditionalists do not test, (*n* = 8)	*It is difficult to approach someone and tell the person to go to clinic to test. They think HIV is death sentence and it is better not to test because if you test you will live with stressor you will be told that the sickness is from makgoma*.
	Health policy: contributions to the second ‘90’ – initiating treatment, (*n* = 1)	*As people living with HIV we lacked knowledge to help us manage our HIV with confidence. Instead some of us exposed ourselves to risk by not always taking the medication from the clinic. After the WWS training we realise HIV is now a chronic illness if you take the right medicine*.
	Health policy: contributions to the third ‘90’ – achieving and maintaining a fully suppressed viral load, (*n* = 13)	*We now live normal lives, we openly come to support group sessions without any fear like before. We are always happy because we have learned that we can manage HIV and live a long positive life with it. We look healthy because we now compete on who takes his or her medication better and the nurses monitor our progress*.
Dimension 3.2: Adjacent acceptable – national scale (NSP)	Reduction in stigma (*n* = 4)	*We now live normal lives. We openly come to support group sessions without any fear like before*.
	Reduction in internalised stigma (*n* = 3)	*I no longer think HIV is the same as dirty blood* [*makgoma*]*. HIV infects people differently to how makgoma infects people, therefore I am no longer confused by the two, like most people in our community. It makes me feel better to know that I do not need to feel bad about makgoma.*
	Increased disclosure (*n* = 4)	*I look healthy because I take my medication every day and when I disclosed to my family they think I am joking*.
	Reduction in denialism (*n* = 2)	*The fear I used to have is gone, I no longer deny my HIV. It helps to know that HIV is different to makgoma*.
Dimension 3.3: Adjacent acceptable – local scale (lifeworlds of the group discussion participants)	Utility of the origins of HIV action theme (*n* = 14)	*In our culture, you have to understand that it is very important for people who are feeling sick to be able to identify the cause of the illness. When people know the cause, then they will take action in ways that are aligned to the cultural belief. For example, some sicknesses require going to the clinic and other illnesses, like makgoma, need a THP*. … *Understanding this difference* [*between the two diseases*] *helps me to remember to take ART*.
		*People in our villages will never stop following or practicing their culture and tradition. What the ‘origins’* [*of HIV action theme*] *does is help us to see that HIV is not part of our tradition. It is a new disease that is different to makgoma. We still respect makgoma, but now we can respect HIV and live a healthy life with it too.*
	Utility of the viral load action theme (*n* = 13)	*I learned about the viral load and the importance of focusing on adhering on my treatment to keep the viral load down.*
	Utility of the chronic not death sentence action theme (*n* = 9)	*Some know their status but they don’t want to disclose to their family. It is difficult to explain that HIV can be treated by ART because it is a natural disease and not makgoma. They do not believe it is a chronic disease that must be treated every day*.
Dimension 3.4: Adjacent acceptable (increasing the influence of the action themes)	Desire by Support Group members to share the information with others (*n* = 11)	*It hurts that many people still don’t know their HIV status and that the virus keep on spreading. It would be better for our community if we could explain that HIV is different to makgoma. I think we must do door to door and share this knowledge because our people are dying.*

Source: Author’s contribution.

The findings indicate that bio-social challenges relating to ending AIDS do exist within the rural communities that the participants were familiar with. This bio-social context represents a deficit situation. The categories that emerged correspond with some of the priority areas identified in the NSP. The findings also indicate that the HIV/makgoma conflation adversely influences the first two ‘90s’ (testing and initiating treatment).

Table 4 provides an overview of the content of the discussions that were coded against the adapted dimensions of the opportunity vacuum model developed by Planing (2017). There are indicators that:

1. Dimension 1: the adjacent feasible (technology) has happened, albeit with minimal use of technology. However, if the basic definition of Dimension 1 is applied – the application of scientific knowledge for practical purposes – it indicates that this criterion is achieved because scientific knowledge (the A-3B-4C-T training) has been integrated into the EAD’s working environment for two years;
2. There were no primary findings relating to Dimension 2: the adjacent viable (economy). However, the integration costs were minimal and by integrating the action themes into existing educational packages, no significant additional costs were incurred (see Limitations, below, for further information), and Insights into Dimension 3, the adjacent acceptable (society) indicates that the pilot has been contributing to 90-90-90 and wellness from multiple perspectives (global, national and local) and that the participants recommend the action themes are shared with other traditionalists living with, or at risk of, HIV (see Table 4).

## Discussion

The Discussion is split into four sections in order to reflect on (1) the utility of the adapted opportunity vacuum model; (2) the findings; (3) the implications of the findings to the current NSP, and (4) the implications of the findings from the perspective of the potential of the pilot to be expanded into a more robust innovation.

### The utility of the opportunity vacuum model

Planing’s (2017) opportunity vacuum model was designed to evaluate the potentials of an idea to be developed into a tangible artefact or service (the process of innovation). Adapting the model as a heuristic to evaluate the potential health related innovation was straightforward and produced pragmatic insights. Problematising Dimension 3, the adjacent acceptable (society), into four sub-dimensions enabled more nuanced insights into the global, national and local relevance of the findings, as well as the group discussion participants’ spontaneous recommendations that the action themes and implications be shared with others, as that added further value during the evaluation. On reflection, Planing’s opportunity vacuum model of innovation, when applied as a heuristic, was found to be fit for purpose and could, with further refinement, be applied to similar evaluations of potential innovations.

### Insights afforded by the re-analysis of the group interviews

#### The ‘exiting’, deficit situation

Table 3 provides indicators that the respondents believed that the existing (prior to the pilot) situation that was generated by the HIV/makgoma conflation represented a deficit condition which was adversely affecting testing and treatment (the first and second ‘90s’) within their communities. Given the estimate that as many as 1.5 million people who are believed to be living with HIV in the provinces where Sotho-Tswana language use is widespread, and are consequently likely to be influenced in variable ways by the HIV/makgoma conflation, Table 2, it is plausible that similar deficit situations exist in indeterminate ways in other areas in central and northern South Africa. This has potentially profound implications for ending AIDS by 2030 because it influences two of the biomedical ‘90s’ in the affected areas and is worthy of further research.

#### Dimension 3.1 of the opportunity vacuum model: adjacent acceptable – global scale (global health policy recommendations)

These findings relate to UNAIDS’ recommended global 90-90-90 strategy that the South African NDoH has adopted. The group discussion findings indicate that the action themes did not contribute to the first ‘90’ (testing) – but the support group members had already tested prior to the integration of the action themes. However, group discussion findings did reveal that some people in their communities do not test because they believe the symptoms are caused by makgoma, not HIV. This makes insights into the first ‘90’ ambiguous and, thus, it is suggested that applying the three actions themes amongst groups of traditionalists who have not tested recently would enable more informed insights.

There was evidence that the action themes have contributed to improving the second and third ‘90s’ (treatment and a fully suppressed viral load), as exemplified in the participant quotations below.

As people living with HIV we lacked knowledge to help us manage our HIV with confidence. Instead some of us exposed ourselves to risk by not always taking the medication from the clinic. After the WWS training we realise HIV is now a chronic illness if you take the right medicine (support group member, Table 4).

We are always happy because we have learned that we can manage HIV and live a long positive life with it. We look healthy because we now compete on who takes his or her medication better and the nurses monitor our progress (support group member, Table 4).

Both are relevant to the NSP and the latter – the last ‘90’ – is especially relevant to the evaluation because of the ‘poor’ rates of adherence ‘due to the use of alternative or traditional medicines’ that have been reported on by NDoH representatives in Limpopo Province (SANAC, 2016, p. 77).

#### Dimension 3.2 of the opportunity vacuum model: adjacent acceptable – national scale (NSP)

The findings indicate that by default – because the South African NDoH has adopted the 90-90-90 strategy – the action themes have contributed to the last two ‘90s’. Other priority areas in the NSP that the pilot has contributed to include: a reduction in stigma and internalised stigma and an increase in disclosure, as indicated in the following participant quotations:

We now live normal lives. We openly come to support group sessions without any fear like before (support group member, Table 4).

I no longer think HIV is the same as dirty blood [makgoma]. HIV infects people differently to how makgoma infects people; therefore I am no longer confused by the two, like most people in our community. It makes me feel better to know that I do not need to feel bad about makgoma (support group member, Table 4).

I look healthy because I take my medication every day and when I disclosed to my family they think I am joking (support group member, Table 4).

These findings are indicators that the pilot has the potential to be applied in other areas associated with high levels of traditionalism in areas where Sotho-Tswana language use is common.

#### Dimension 3.3 of the opportunity vacuum model: adjacent acceptable – local scale (lifeworlds of the group discussion participants)

The findings indicate that each action theme contributed to reducing the HIV/makgoma conflation that had been adversely influencing the wellness of the support group members.

In our culture, you have to understand that it is very important for people who are feeling sick to be able to identify the cause of the illness. When people know the cause, then they will take action in ways that are aligned to the cultural belief. For example, some sicknesses require going to the clinic and other illnesses, like makgoma, need a THP. … . Understanding this difference [between the two diseases] helps me to remember to take ART (support group member, Table 4).

I learned about the viral load and the importance of focusing on adhering on my treatment to keep the viral load down (support group member, Table 4).

It is difficult to explain that HIV can be treated by ART because it is a natural disease and not makgoma. They do not believe it is a chronic disease that must be treated every day (support group member, Table 4).

It is not possible to comment whether the action themes’ utility is derived from the action themes functioning independently, or whether it is the combination that has had an impact. This is worthy of further research. Nevertheless, enabling the support group members to problematise the HIV/makgoma conflation in ways that are culturally acceptable is extremely relevant because culture is often overlooked in these types of biomedical contexts (Rubincam, Lacombe-Duncan, & Newman, 2016). This is emphasised in the comment:

People in our villages will never stop following or practicing their culture and tradition. What the ‘origins’ [of HIV action theme] does is help us to see that HIV is not part of our tradition. It is a new disease that is different to makgoma. We still respect makgoma, but now we can respect HIV and live a healthy life with it too (support group member, Table 4).

#### Dimension 3.4 of the opportunity vacuum model: adjacent acceptable (increasing the influence of the action themes)

The support group members did indicate that they believe that the action themes should be shared with other people either at risk of HIV, or living with HIV.

It would be better for our community if we could explain that HIV is different to makgoma. I think we must do door to door and share this knowledge because our people are dying (support group member, Table 4).

Recent discussions with the EAD team leader also indicated that another support group that WWS does not work with has adopted the action themes into their activities. This happened through word of mouth referrals from WWS support group members.

### The implications of the findings to the current NSP

Both UNAIDS and SANAC acknowledge that biomedical innovations have enabled 90-90-90 to become realisable and that bio-social innovations can contribute to, or reinforce, the social practices required to end AIDS among diverse communities. It is clear that the dominant supply-side of the biomedical innovation that has been labelled 90-90-90 is science. However, as Planing’s (2017) opportunity vacuum model of innovation underscores, innovation is as much about the sociality of the uptake (demand-side) of the innovation as it is about the supply-side.

Goal 3 of the NSP identifies populations who are more at risk than the general population which are labelled as ‘key and vulnerable populations’ (SANAC, 2017, p. 23). Traditionalists, or people who are influenced by medical pluralism, are not listed within the key and vulnerable populations identified in Goal 3. However, a belief is not a population, *per se*. A belief is an influence that can cut across every key and vulnerable population that the NSP cites in variable ways. Consequently, it is suggested that integrating the action themes into existing strategies that target key or vulnerable groups who live in areas where Sotho-Tswana language use is common, could be a value adding addition because it is plausible that medical pluralism influences all of the key and vulnerable populations in indeterminate ways.

### Implications of the findings from the perspective of increasing the influence of the action themes

Applying Planing’s (2017) opportunity vacuum model as an evaluation heuristic has facilitated pragmatic insights into the potential of the intangible idea (the pilot) that is now moving towards becoming a tangible artefact, or service (the outputs). Qualitative indicators suggest that the three action themes have contributed to the second and third ‘90’ and have also contributed to other priority areas identified in the NSP.

At this stage, the contribution of the action themes to 90-90-90 is ambiguous in terms of the underlying driving mechanisms. However, following Rubincam’s (2015, p. 3) argument that treatment preferences tends to reflect a ‘street level epistemology of trust’ in particular models of health care, it is suggested that the utility of the action themes – which were identified and developed by community-based practitioners – has been to facilitate a shift in levels of trust from a traditionalist perspective towards a biomedical perspective, without dismissing the traditionalist position as being one of quackery. In the context of post-colonial sub-Saharan Africa, an intervention that does not denigrate, or ‘other’, African cultural traditions, whether medical, or otherwise, is relevant because ‘African countries and peoples continue to be deeply affected by the colonial past and, linked to this, suspicion and distrust are in abundance’ (Mkhwanazi, 2016, p. 201). Despite the above, the pilot is restricted to a localised area in the Waterberg district of Limpopo Province and it is necessary to replicate the pilot in other areas to confirm the potentials of the intervention to contribute more substantially to 90-90-90.

## Policy implications

The NSP has committed to supporting evidence-informed contributions to achieving 90-90-90 using implementation science techniques. The potential innovation that has been reported on has been developed by a community-university partnership that has focused exclusively on the day to day realities of traditionalists affected by HIV in rural communities in Limpopo Province, South Africa. The findings suggest that the outputs from the pilot are contributing to the NSP at a localised scale and contain the potentials to be developed into a tangible innovation.

In South Africa this potential innovation is relevant because (1) there are significant numbers of people who are influenced by medical pluralism and it has been demonstrated that medical pluralism can have, albeit unintended, adverse health related outcomes, and (2) there have been very few (if any) substantial documented interventions designed to reduce the unintended adverse impacts of medical pluralism on health and wellness in the context of HIV and AIDS (Leclerc-Madlala et al., 2016). Consequently, the first policy suggestion is that both regional and national policy makers could consider (1) recognising that medical pluralism as an influential force and prioritise, or acknowledge, this in HIV and AIDS strategies, and (2) proactively support research initiatives designed to respond to the unintended, yet adverse, influence of medical pluralism on the trajectory of the HIV epidemic.

These suggestions are aligned with (1) UNAIDS’ (2014a, p. 18) recommendation that it is necessary to step beyond the parameters of business as usual as a mechanism to overcome the ‘different types of HIV and AIDS epidemics which vary considerably within and between countries and regions …  [by] focusing on the locations and populations where risk is greatest’ and (2) the statement by SANAC (2017, p. 21) that the ‘NSP encourages the roll-out of innovative approaches to increase treatment uptake and improve treatment outcomes’.

The second issue is a policy endorsement which reflects the comment by SANAC (2017, p. xii) that it is necessary to ensure that no ‘groups [are] left behind’ during the process of ending AIDS. The expression ‘left behind’ was first coined by UNAIDS in 2014 when they argued that 12 populations had been ‘left behind’ and subsequently committed almost one third of the Gap Report, pages 119-279, to ‘describe the struggles they [the 12 populations] face, why they have been left behind and how to close the gap’ (UNAIDS, 2014b, p. 118). Granted, key populations who have been ‘left behind’ are high priority. However, at a different level of abstraction, the value systems, such as medical pluralism and the commensurate ‘street level epistemology[ies] of trust’, that influence these populations are also relevant. This level of granularity is far more ambiguous, difficult to measure and is mostly erased from policy documents, yet time and again it is peoples’ value systems – contra technically defined ‘populations’ – that discretely influence health related outcomes. Consequently, the commitment by SANAC (2017, p. 50) to prioritise ‘[s]ocial and anthropological research’ is endorsed.

The third issue is also a policy endorsement, on a more localised scale. Policy makers could contribute to improving the visibility of home-grown, culturally sensitive bio-social initiatives that are moving along the intangible idea towards a tangible artefact continuum in the context of health. This would contribute to enabling the movement of ideas towards becoming established, indigenous innovations that make sense to the people that the innovation is designed to benefit – as has been suggested by multiple contexts (Burman, 2018; Chilisa, 2011; Ntseane & Chilisa, 2012). In the context of ending AIDS, SANAC (2017, p. 47) has committed to ‘optimising multidisciplinary strategies to deliver validated interventions’. It is hoped that this will include home-grown, indigenised strategies to reduce the unintended consequences of medical pluralism on the trajectory of the South African HIV and AIDS epidemic.

## Limitations and further research

The primary limitation is that these findings are qualitative. Further mixed method research is required to substantially improve the trustworthiness of the findings. The second limitation relates to cost implications. The introduction of the action themes into the existing work of the EAD has been low cost. The initial start-up funds (the research grant) were used to pay for the training events, travel and monitoring and evaluation. However, because the community partner identified the action themes that made sense to them in their working environment and integrated them into existing programmes, the longer term (day to day) costs are negligible. With hindsight it would have been preferable to document these costs from the outset. The third limitation is that the research method is based on a theoretical heuristic – the opportunity vacuum model – that is not validated. Nevertheless, the purpose of the work was for the partnership to evaluate the innovative potentials of the pilot prior to committing to further investment. From that perspective, the opportunity vacuum model was found to be fit for purpose, despite the limitation of the exclusive use of qualitative findings. The fourth limitation is that the pilot is an isolated community-university partnership. Future development of the pilot intervention should be more inclusive of multi- and/or transdisciplinary community-university partnerships.

## Conclusion

Both UNAIDS and SANAC acknowledge the need for bio-social innovation within the changing HIV and AIDS environment if the 90-90-90 goals are to be achieved. The unintended consequences of medical pluralism on the HIV and AIDS epidemic have been documented since 2004, but to date, very few (if any) sustainable interventions to counter the influence of medical pluralism on HIV and AIDS have been documented. Using an adapted version of the opportunity vacuum model developed by Planing (2017), this reflection aimed to evaluate the potential of a community-university pilot project to be developed into a bonafide innovation. The application of the opportunity vacuum model of innovation as an evaluation heuristic was found to be both pragmatic and straightforward. The findings indicate there (1) the pilot has contributed to 90-90-90 in multiple ways, and (2) there are sufficient global, national and local indicators that the pilot has the potentials to be developed into an innovation that could accelerate movement towards achieving 90-90-90 in Sotho-Tswana language areas that are associated with high levels of traditionalism. Based on the above, it is believed that the pilot represents a nascent innovation which is worth nurturing.
